# Use of skincare products and risk of cancer of the breast and endometrium: a prospective cohort study

**DOI:** 10.1186/s12940-019-0547-6

**Published:** 2019-12-03

**Authors:** Charlotta Rylander, Marit B. Veierød, Elisabete Weiderpass, Eiliv Lund, Torkjel M. Sandanger

**Affiliations:** 1 Department of Community Medicine, UiT The Arctic University of Norway, Tromsø, Norway; 2 Oslo Centre for Biostatistics and Epidemiology, Department of Biostatistics, Institute of Basic Medical Sciences, Faculty of Medicine, University of Oslo, Oslo, Norway; 3 International Agency for Research on Cancer, World Health Organization, Lyon, France; 4 Department of Research, Cancer Registry of Norway, Institute of Population-Based Cancer Research, Oslo, Norway; 5 NILU, FRAM-High North Research Centre for Climate and Environment, Tromsø, Norway

**Keywords:** Skin care, Body lotion, Hand cream, Facial cream, Personal care products, Cancer, Breast, Endometrial, Cohort, Carcinoma

## Abstract

**Background:**

Concerns have been raised that extensive use of personal care products that contain endocrine disrupting compounds increase the risk of hormone sensitive cancers.

**Objective:**

To assess the effect of skincare product use on the risk of pre- and postmenopausal breast cancer, estrogen receptor positive (ER+) and negative (ER-) breast cancer and cancer of the endometrium.

**Methods:**

We used data from 106,978 participants in the population-based Norwegian Women and Cancer cohort. Participants were categorized into non-, light, moderate, frequent and heavy users of skincare products based on self-reported use of hand and facial cream and body lotion. Cancer incidence information from the Cancer Registry of Norway was linked to individual data through the unique identity number of Norwegian citizens. Multivariable Cox proportional hazard regression was used to assess the effect of skincare product use on the risk of cancer of the breast and endometrium. We used multiple imputation by chained equations to evaluate the effect of missing data on observed associations.

**Results:**

We found no associations between use of skincare products and incidence of premenopausal breast cancer (frequent/heavy versus non−/light use: hazard ratio [HR] =1.10, 95% confidence interval [CI]: 0.92–1.32), postmenopausal breast cancer (heavy versus light use: HR = 0.87, 95% CI: 0.65–1.18, frequent versus light use: HR = 0.97, 95% CI: 0.88, 1.07) or endometrial cancer (frequent/heavy versus non−/light use: HR = 0.97, 95% CI: 0.79–1.20). Use of skincare products did not increase the risk of ER+ or ER- breast cancer and there was no difference in effect across ER status (0.58 ≤ p_heterogeneity_ ≤ 0.99). The magnitude and direction of the effect estimates based on complete case analyses and multiple imputation were similar.

**Conclusion:**

Heavy use of skincare products, i.e. creaming the body up to two times per day during mid-life, did not increase the risk of cancer of the breast or endometrium.

## Background

Use of skincare products such as body lotion, facial cream and hand cream is common among Norwegian women [1]. Several components of these personal care products (PCPs) are classified as known or suspected endocrine disruptors (EDs), i.e., compounds that are able to interfere with the endocrine function in humans and wildlife [2]. EDs are easily absorbed by the skin into the central blood circulation after dermal application, and have been detected in various concentrations in human blood, urine and breastmilk [3]. Concerns have therefore been raised whether extensive use of cosmetics and skincare products could increase the risk of hormone related cancer, for instance breast cancer [4].

Among frequently used EDs in PCPs are phthalates, ultraviolet (UV) filters, triclosan and parabens [5]. Phthalates are also commonly used as softeners in consumer products such as food packaging material, children’s toys and building material [6] and have been suggested to interfere with the testosterone production or action [2]. Recently, phthalates have shown anti-estrogenic effect in breast cancer cell lines [7] and induced cell proliferation in normal breast cells [8]. However, recent epidemiological studies reported no significant association between phthalate exposure and breast cancer [9, 10].

UV-filters are a large group of compounds used as constituents in sunscreen as they are able to absorb UV-radiation. They are also included in other PCPs to increase shelf-life [2], and have been detected in human urine [11, 12], breastmilk [13] and breast tissue [14]. Many UV-filters exert estrogenic activity in in vivo*/*in vitro experiments [15], however epidemiological studies of endocrine disrupting effects of UV-filters are rare. Triclosan is primarily used as an antimicrobial agent in soap, toothpaste, cosmetics and pharmaceuticals and has shown endocrine disrupting properties in experimental settings [2]. Prenatal triclosan concentrations was recently reported associated with earlier menarche in American girls, whereas there was no effect of prenatal or peripubertal concentrations of the UV filter Benzophenone-3 [16].

Parabens are alkyl esters of *p*-hydroxybenzoic acid which is naturally occurring in several plants and berries, such as blueberries, strawberries, red and white currants [17, 18]. For decades, methyl-, ethyl, propyl- and butyl-paraben were the most frequently used preservatives in skincare products due to their antimicrobial properties, low toxicity, cost and weak sensitizing properties. From the beginning of this century, restrictions on the use of parabens in consumer products have been implemented within the European Union as a result of scientific reporting of weak estrogenic activity of parabens. Several in vitro studies have shown that parabens are able to bind to the estrogen receptor and stimulate proliferation within human breast cancer cell lines [19, 20], also in concentrations similar to what have been detected in human breast cancer cells [21] and in human breast tissue [22]. Subcutaneous exposure to butyl and isobutyl-paraben has also been linked to uterus enlargement in rodents as a result of estrogenic activity [23] and peripubertal concentrations of methyl- and propoyl-paraben have been associated with measures of pubertal timing in girls [16]. In our previous study, we showed that women who cream their body once daily or more had elevated plasma concentrations of methyl-, ethyl- and propyl- parabens [24]. Other studies have found similar results [25], and there are reason to believe that women who use skincare products frequently experience higher body burdens of other EDs as well, for instance phthalates, UV filters and triclosan [12, 26].

Epidemiological evidence of the effects of cosmetics, skincare products or constituents of PCPs on hormone sensitive cancers, such as cancer of the breast and endometrium, is sparse. A recent cohort study reported 13% increased risk of breast cancer by frequent use of skincare products and 15% increased risk by frequent use of beauty products [27]. Parada et al. found positive associations between paraben concentrations in urine (methyl- propyl and ∑parabens) and prevalent breast cancer, and negative, non-significant associations with breast cancer mortality [28]. Out of four case-control studies of antiperspirant use and breast cancer risk, two reported no effect [29, 30] and one found an increased risk of breast cancer by use of underarm cosmetics [31]. The forth study reported an association between earlier age at breast cancer diagnosis and longer duration of deodorant use and underarm shaving [32]. Use of skin lighteners has recently been reported not being associated with breast cancer [33]. Thus, given the widespread use of skincare products and the potential estrogenic effects of product constituents, there is a clear lack of epidemiological studies addressing the effect of skincare product use on hormone sensitive cancers. In this population-based study, we aimed to investigate the effect of skincare product use on the risk of pre- and postmenopausal breast cancer, estrogen receptor positive (ER+) and negative (ER-) breast cancer and cancer of the endometrium.

## Methods

### Study design, participants and sub-samples

The Norwegian Women and Cancer study (NOWAC) is a national representative cohort study initiated in 1991 with the aim of exploring associations between lifestyle and cancer among Norwegian women [34]. Women aged 30–70 years were randomly selected from the National Registry and invited to participate in the study through a mailed invitation letter to their home address that also included a detailed questionnaire. Women that agreed to participate have been followed-up regularly with consecutive questionnaires. Since the initiation of the study, the cohort has been expanded several times and includes today about 172,000 women. All participants have answered between one and four questionnaires regarding their current health status and lifestyle factors. The questionnaires have been distributed in waves and the level of details has varied between the questionnaires. The external validity of NOWAC has been studied extensively and been found satisfactory and without selection bias [35].

**Fig. 1:**
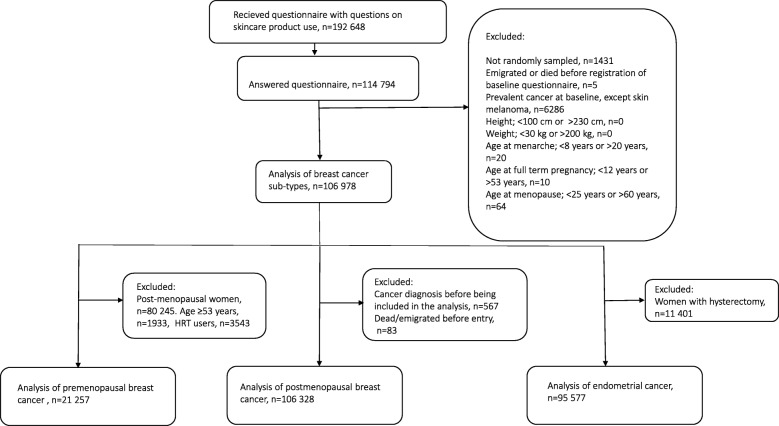
Flow chart of inclusion and exclusion criteria. The Norwegian Women and Cancer cohort 2003–2011

Questionnaires that included questions about usage frequencies of hand cream, facial cream and body lotion were mailed to 192,648 women, of which 114,794 responded. The questions regarding usage of skincare products were the same in all questionnaires. We excluded 1431 women that belonged to a certain wave of recruitment that was not randomly sampled and 5 women that died or emigrated before their baseline questionnaire were registered. Additionally 6286 women were excluded as they were diagnosed with cancer (except non-melanoma skin cancer, International Statistical Classification of Diseases, Injuries and Causes of Death Revision 10 code [ICD-10]: C44) prior to answering the questionnaire. Women reporting implausible values on height, weight, age at menarche, age at first full term pregnancy and age at menopause were also excluded from the study (*n* = 94). Thus, the final study sample used for studying the associations between skincare product use and breast cancer subtypes included 106,978 women (Fig. 1).

After excluding 85,721 women that were either postmenopausal (*n* = 80,245), used menopausal hormone therapy (MHT, *n* = 3543) or were ≥ 53 years at baseline (*n* = 1933), the study sample for premenopausal breast cancer included 21,257 women (Fig. 1). Women were considered postmenopausal if they stated that their menstrual bleeding had stopped or reported use of MHT if they were ≥ 53 years. This cut-off is based on the definition used in the Million Women Study [36] and has been validated against plasma concentrations of sex hormones in NOWAC [37].

The study sample for postmenopausal breast cancer included 106,328 women (Fig. 1). Women that were not categorized as postmenopausal at baseline were included in the study from the age of 53 years or the age of reported menopause. Due to this left truncation, we excluded 567 women that were diagnosed with cancer and 83 that died or emigrated before the start of follow-up. The study sample for endometrial cancer included 95,577 women. Here, we excluded women that reported hysterectomy (*n* = 11,401).

### Measures of skincare products use and included covariates

In the NOWAC questionnaires, women were asked to record how often they used skincare products such as body lotion, hand cream and facial cream (never/seldom, 1–3 times/month, 1 time/week, 2–4 times/week, 5–6 times/week, 1 time/day and ≥ 2 times/day). The recorded frequencies were converted to percentage body surface covered with cream per day. Use of hand cream, facial cream and body lotion once per day corresponded to 100% of body surface covered in cream per day. Calculated percentages were later categorized into five groups; non-users (0–0.001%), light users (0.002- < 35.0%), moderate users (35.0- < 65.0%), frequent users (65.0- < 115.0%) and heavy users (115.0–200%), where 200% corresponded to using hand cream, facial cream and body lotion twice per day. The conversion is described in detail in Aniansson et al. [1] and has also been used previously to assess the correlation between skincare product use and plasma concentrations of parabens [24].

Information on the women’s age was extracted from the National Registry and the other covariates from the baseline NOWAC questionnaire. Education was categorized based on years of completed schooling corresponding to secondary school (< 10 years), high school (10–12 years) and higher education (> 12 years). Body mass index (BMI) was calculated from self-reported body weight (kg) and height (m), and categorized as under−/normal weight (< 25.0 kg/m^2^), overweight (25.0–29.9 kg/m^2^) or obese (≥30 kg/m^2^). The women’s menopausal status (premenopausal, perimenopausal, postmenopausal, unknown) was determined by reported regular menstrual bleeding or not, use of MHT and the women’s age [36]. Age at menarche was categorized into three groups (≤12 years, 13–14 years, ≥15 years) and age at first full term pregnancy and parity were combined into one variable (nullipara, < 30 years at first full term pregnancy and unipara, ≥30 years and unipara, < 30 years and multipara, ≥30 years and multipara) in the statistical analysis. Use of oral contraceptives (OCs) and MHT were categorized in two ways; never/former/current was used for the breast cancer analysis, and the never/ever categorization for endometrial cancer. Use of intrauterine device (IUD) was also assessed as never/ever use. Smoking status was categorized as never, former and current smoker. Physical activity was recorded on an ordinal 10-point scale and categorized as low (1–4), moderate (5–6) and high (≥7). Alcohol consumption was recorded by a food frequency questionnaire and used in continuous scale.

### Cancer incidence, tumor receptor status, death and emigration

The NOWAC study receives annual updates from the Cancer Registry of Norway in order to identify study participants diagnosed with cancer during the preceding year. Information on date of diagnosis and hormone receptor status for the breast cancer cases are also included. In the present study, women diagnosed with a first primary invasive malignant neoplasm of the breast (ICD-10: C50) and uterus (ICD-10: C54) were identified. Endometrial cancer cases were identified from morphological codes 8380, 8382, 8480, 8481, 8560, 8570, 8020, 8041, 8045, 8255, 8310, 8441, 8460 or 8323 from the International Classification of Disease for Oncology ICD-O-3. Information about deaths and emigration was extracted from the Causes of Death Registry and the National Registry. End of follow-up was December 31, 2016.

We followed the official Norwegian thresholds for classification of hormone receptor status [38]. From February 2012 and onwards, a tumor was classified as ER- if it displayed < 1% reactivity, whereas prior to February 2012, the threshold for ER- was < 10%. Accordingly, tumors classified as ER+ displayed ≥10% reactivity prior to February 2012 and ≥ 1% after. The change in threshold was due to changes in clinical practice at Norwegian hospitals.

### Statistical analysis

We investigated the associations between skincare product use and the risk of breast and endometrial cancer using Cox proportional hazard regression with age as the time scale. Entry time was age at answering the questions on skincare products, and exit time was age at cancer diagnosis, death, emigration or end of follow-up, whichever occurred first. In the analysis of premenopausal breast cancer, women were censored at the age of 53 or age at reported menopause if that occurred prior to cancer diagnosis, death, emigration or end of follow-up. Due to few cancer cases among the non- and heavy users of skincare products, we used three exposure groups (non−/light, moderate, frequent/heavy) in the analysis of premenopausal breast cancer, endometrial cancer and the subtype-specific analysis of breast cancer. Light users (or non−light users) were used as the reference group in all analysis. We used the “purposeful selection method” described by Hosmer and Lemeshow [40] to evaluate which covariates to include in the final multivariable models. Covariates included in each regression model are listed in the table footnotes. To test for linear trend, we replaced the group identifier with the median use of % skin covered in cream per day per group, and included that variable in the multivariable models. Additionally, to model the relationship between cancer risk and use of skincare products in continuous scale and to allow for non-linear effects, we fitted regression models with natural cubic spline transformations (4 knots) of the exposure variable (% skin creamed per day). The knots were placed at equally spaced percentiles. We evaluated non-linearity by testing the null hypothesis of the second and third spline coefficients jointly equalled zero. We assessed effect modification by MHT use for postmenopausal breast cancer, but did not evaluate any other interactions, due lack of statistical power. Departures from the proportional hazard assumption were assessed by inspection of the Schoenfeldts residuals. Participants with missing values of included covariates were excluded from the complete case analysis.

To assess differences in sub-type specific breast cancer risk, we used Cox proportional hazard regression for ER+ and ER- separately, where women who were diagnosed with another breast cancer subtype, were censored at the time of diagnosis [41]. We tested for heterogeneity in associations between subtypes by a chi-squared (contrast) test [42].

In the complete case analysis of premenopausal breast cancer, 92% of the observations were included. Corresponding proportions for postmenopausal breast cancer, ER+, ER- breast cancer and endometrial cancer were 83, 84, 90 and 69%, respectively. To evaluate the effect of missing information on the observed results, we used multiple imputation by chained equations to obtain 20 imputed datasets with complete observations for each outcome. The hazard ratios (HRs) estimated on the imputed data sets were pooled together using Rubin’s rule to obtain valid statistical inferences [43].

Several additional analyses were performed. We assessed the associations between recorded usage frequencies of body lotion (categorized as never/seldom, 1–4 times/month, 2–6 times/week and 1- ≥ 2 times/day) and breast and endometrial cancer incidence. Furthermore, we summarized the usage frequencies of skincare products (scores from 0 [no use of any of skincare products] to 18 [use of hand cream, facial cream and body lotion ≥2 times/day]; categorized as low [0–5], moderate low [6–10], moderate high [11–14] and high [15–18]) and studied the associations with risk of breast and endometrial cancer.

All *P*-values were two-sided and a 5% level of significance was used. The statistical analysis was conducted using Stata, version 15.1 (StataCorp, College Station, TX, USA).

## Results

In the total study sample of 106,978 women, mean (standard deviation [SD]) age at enrollment was 54.7 (4.8) years. During the mean follow-up time of 10.7 (2.6) years, 3408 women were diagnosed with breast cancer and 681 with endometrial cancer. Mean age at breast and endometrial cancer diagnosis were 60.1 (5.5) and 61.0 (5.2) years, respectively.

Age at enrollment was similar in all categories of skincare product use. Compared to light users, a larger proportion of women with under−/normal weight and postmenopausal status were found among the heavy users. Further, heavy users were slightly younger when they had their first child and a larger proportions had < 3 children compared to the light users. Heavy users were also more frequent current and former users of OC, MHT and cigarettes, more physically active and reported higher intake of alcohol than the light users (Table 1).

**Table 1:** Characteristics of study participants (*n* = 100,436) ^a^ by categories of skincare product use

Characteristics	Non-users	Light users	Moderate users	Frequent users	Heavy users
n	1840	36,598	25,718	34,203	2077
% skin creamed per day	0–0.001	0.002- < 35.0	35.0- < 65.0	65.0- < 115.0	115.0–200
Incident cases of breast cancer (n)	65	1176	801	1088	58
Breast cancer subtype, % ^b^					
ER+	83.1	83.8	85.3	84.1	84.5
ER-	15.4	13.9	12.1	12.9	15.5
Incident cases of endometrial cancer (n)	15	261	141	216	9
Age at enrollment (years), mean ± SD	54.8 ± 5.0	54.3 ± 4.8	54.9 ± 4.8	54.8 ± 4.8	54.8 ± 4.8
Education (years), %					
< 10	27.2	15.9	17.0	15.4	15.1
10–12	30.4	32.1	36.4	36.1	34.8
> 12	42.4	51.9	46.5	48.5	50.1
BMI, %					
Under−/Normal weight	36.6	50.7	56.7	60.5	66.4
Overweight	33.4	34.8	32.7	30.7	25.7
Obese	30.0	14.5	10.6	8.8	7.9
Menopausal status at enrollment, %					
Pre	14.2	15.7	13.0	13.4	13.4
Peri	9.2	10.3	9.5	9.4	8.9
Post	72.4	68.6	72.2	71.5	72.1
Unknown	4.2	5.4	5.2	5.7	5.6
Age at menarche, mean ± SD	13.2 ± 1.5	13.3 ± 1.4	13.3 ± 1.4	13.2 ± 1.4	13.2 ± 1.5
Age at first full term pregnancy, mean ± SD	23.9 ± 4.6	24.4 ± 4.7	24.0 ± 4.4	24.0 ± 4.4	23.9 ± 4.4
Parity, %					
0	10.3	10.2	9.3	9.2	10.4
1–2	47.3	53.3	56.7	59.4	58.3
≥3	42.4	36.6	33.9	31.4	31.3
OC use, %					
Never	48.2	40.3	38.1	36.0	36.4
Former	51.3	59.2	61.4	63.4	62.8
Current	0.5	0.5	0.5	0.6	0.8
MHT use, %					
Never	69.7	63.7	56.7	53.7	50.8
Former	21.4	23.5	27.4	27.2	27.3
Current	8.9	12.8	16.0	19.0	21.8
IUD use, %					
Ever	7.7	11.6	11.9	12.2	12.7
Smoking, %					
Never	37.4	36.0	32.4	31.0	31.6
Former	30.9	40.8	43.9	45.4	43.6
Current	31.7	23.2	23.7	23.6	24.8
Physical activity, %					
Low	37.6	24.8	19.8	16.5	14.6
Moderate	34.8	41.8	42.4	38.9	32.6
High	27.6	33.4	37.8	44.6	52.8
Alcohol intake (g/day), mean ± SD	2.7 ± 4.2	3.7 ± 4.3)	4.1 ± 4.2	4.4 ± 4.3	4.2 ± 4.4

*SD* standard deviation, *ER* estrogen receptor, *BMI* body mass index, *OC* oral contraceptive, *MHT* menopausal hormone therapy, *IUD* intrauterine device

^a^6542 participants had missing values on skincare product use

^b^Percentage of ER+/ER- subtypes does not add up to 100 due to missing values on hormone receptor status

**Fig. 2:**
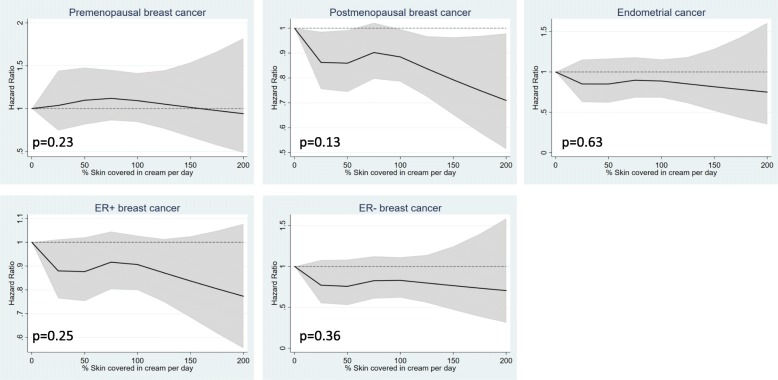
Associations between skin care product use and pre- and postmenopausal cancer, endometrial cancer and estrogen receptor positive (ER+) and negative (ER-) breast cancer. Skin care product use is modelled in continuous scale using restricted cubic spline transformations of “% skin covered in cream per day” with 4 knots. The *p*-value corresponds to the null hypothesis that the regression coefficient for the second and third spline transformations equaled zero, i.e., a test of non-linearity

Frequent/heavy users of skincare products did not experience increased risk of premenopausal breast cancer (HR = 1.10, 95% confidence interval [CI]: 0.92–1.32), postmenopausal breast cancer (heavy versus light use: HR = 0.87, 95% CI: 0.65–1.18, frequent versus light use: HR = 0.97, 95% CI: 0.88, 1.07) or endometrial cancer (HR = 0.97, 95% CI: 0.79–1.20) compared to the non−/light users. Nor did the moderate users, and there was no linear trend across effect estimates (0.27 ≤ p_trend_ ≤ 0.63) In fact, moderate use of skincare products was associated with 23% decreased risk of endometrial cancer (HR = 0.77, 95% CI: 0.61, 0.98) (Table 2). The regression models with natural cubic spline transformations of %skin covered in cream per day, displayed no increased risk of pre- or postmenopausal breast cancer or endometrial cancer by increasing use of skincare products. However, an inverse association between skincare product use and postmenopausal breast cancer risk was suggested (Fig. 2 and Additional file 1). Further, there was no evidence of an interaction between skincare product use and MHT use in relation to postmenopausal breast cancer risk (p _interaction(Wald test)_ = 0.44).

**Table 2:** Associations between skincare product use and incidence of pre−/postmenopausal breast and endometrial cancer

User groups of skincare products per cancer type	n	Cancer cases	Age-adjusted HR (95% CI)	Multivariable HR (95% CI)	p_trend_
Premenopausal breast cancer^a^					
Non−/Light users	7988	238	1.00	1.00	0.56
Moderate users	4642	146	1.05 (0.86,1.30)	1.05 (0.85,1.29)	
Frequent/heavy users	7215	237	1.11 (0.93,1.33)	1.10 (0.92,1.32)	
Postmenopausal breast cancer^b^					
Non-users	1549	49	1.12 (0.84,1.49)	1.14 (0.85,1.52)	0.27
Light users	32,379	922	1.00	1.00	
Moderate users	22,762	656	1.00 (0.90, 1.10)	0.99 (0.89,1.09)	
Frequent users	29,874	854	0.99 (0.90, 1.09)	0.97 (0.88,1.07)	
Heavy users	1752	45	0.89 (0.66, 1.20)	0.87 (0.65,1.18)	
Endometrial cancer^c^					
Non−/Light users	25,774	213	1.00	1.00	0.63
Moderate users	16,556	98	0.70 (0.55,0.88)	0.77 (0.61,0.98)	
Frequent/heavy users	23,156	163	0.83 (0.68,1.02)	0.97 (0.79,1.20)	

*HR* hazard ratio, *CI* confidence interval

^a^Multivariable adjusted for maternal breast cancer history and alcohol intake

^b^Multivariable adjusted for body mass index, use of menopause hormone therapy, age at first birth and parity combined, maternal breast cancer history, physical activity and alcohol intake

^c^Multivariable adjusted for body mass index, use of oral contraceptives, use of intrauterine device, smoking and education

The subtype-specific breast cancer analyses suggested no increased risk of ER+ or ER- breast cancer by moderate or frequent/heavy use of skincare products and no difference in effect by estrogen receptor status (0.58 ≤ p_heterogeneity_ ≤ 0.99, Table 3).

**Table 3:** Associations between skincare product use and incidence of ER+ and ER- breast cancer

			ER + breast cancer^a^				ER- breast cancer^b^		
User group of skincare products	n	Cancer cases	Age-adjusted HR (95% CI)	Multivariable HR (95% CI)	n	Cancer cases	Age-adjusted HR (95% CI)	Multivariable HR (95% CI)	p_heterogeneity_^c^
Non−/light users	33,309	883	1.00	1.00	35,090	157	1.00	1.00	
Moderate users	22,411	585	0.98 (0.88,1.08)	0.98 (0.89,1.09)	23,475	92	0.89 (0.69,1.15)	0.91 (0.7,1.18)	0.58
Frequent/heavy users	35,784	948	0.99 (0.90,1.09)	0.99 (0.90,1.08)	37,896	159	0.95 (0.76,1.19)	0.99 (0.79,1.23)	0.99

*HR* hazard ratio, *CI* confidence interval; ER: estrogen receptor

^a^Multivariable adjusted for body mass index, smoking, age at first birth and parity combined, alcohol intake, physical activity, menopausal status, maternal breast cancer history and use of menopause hormone therapy

^b^Multivariable adjusted for physical activity and maternal breast cancer history

^c^Test of difference in effect by breast cancer subtypes

The magnitude and direction of the effect estimates based on complete case analyses and multiple imputation were similar, except for the lack of inverse association between moderate skincare product use and risk of endometrial cancer (Additional files 2 and 3). Thus, missing values did not bias the observed associations in the complete case analyses.

In the additional analyses, we found no significant associations between usage frequencies of body lotion and the risk of pre- or postmenopausal breast cancer, endometrial cancer, ER+ or ER- breast cancer (body lotion: 0.30 ≤ p_trend_ ≤ 0.55) (Additional file 4). However, there was a significant inverse trend for usage frequencies of skincare products combined and postmenopausal breast cancer and ER+ breast cancer (0.02 ≤ p_trend_ ≤ 0.046), although we did not find significantly reduced HRs for any of the categories (postmenopausal breast cancer: moderate low versus low use: HR = 1.01, 95% CI: 0.88,1.17; moderate high versus low use: HR = 0.94, 95% CI: 0.81,1.08; high versus low use: HR = 0.89, 95% CI: 0.77,1.03; ER+ breast cancer: moderate low versus low use: HR = 1.07, 95% CI: 0.91,1.24; moderate high versus low use: HR = 0.95, 95% CI: 0.82,1.11; high versus low use: HR = 0.93, 95% CI: 0.80,1.09) (Additional file 5). Endometrial cancer and ER- breast cancer were not associated with usage frequencies of skincare products combined (0.20 ≤ p_trend_ ≤ 0.45) (Additional file 5).

## Discussion

In this large and national representative cohort of women in Norway, we found no evidence of increased risk of pre- or postmenopausal breast cancer by use of skincare products. Nor did we observe evidence of increased risk of ER+ or ER- breast cancer or endometrial cancer. We have previously reported that frequent and heavy users of skincare products in the NOWAC study experience elevated plasma concentrations of methyl-, ethyl and propyl-parabens, compounds that exert endocrine disrupting properties and have been linked to breast cancer in in vivo and in vitro studies, as well in a recent case control study [28, 44, 45]. We can now conclude that these women did not experience increased risk of hormone sensitive cancers. In fact, an inverse relationship between skincare product use modelled in continuous scale with restricted cubic splines and postmenopausal breast cancer risk was observed, which could be a chance finding or a result of residual confounding.

To this date, our study is the largest prospective cohort study assessing the effect of skincare product use on cancer risk. Our results may seem to contrast Taylor et al. who recently reported increased risk of breast cancer by frequent use of skincare products among non-Hispanic white women from the United States [27]. However, that study included both in situ and invasive breast cancer and stratified analyses displayed no significant association between frequent use of skincare products and invasive breast cancer, which is in correspondence with our results. Further, the authors found no significant associations between skincare product use and ER+ or ER- breast cancer which is in line with our findings. Notably, Taylor and colleagues included nine different skincare products (face cream, cleansing cream, anti-aging cream, foot cream, body lotion, hand lotion, petroleum jelly, and two different talcum powders) in comparison to three in the present study, which could explain differences in results. In the analyses stratified by breast cancer type, Taylor et al. observed a borderline significant increased risk of in situ breast cancer among frequent users of skincare products compared to infrequent users, which may suggest that the association with skincare product use may be different in in situ and invasive breast cancer. Additionally, skincare product constituents in the United States and in Europe may vary due to differences in regulations, which could also affect the results.

Recently, Parada et al. reported positive associations between urinary concentrations of parabens and breast cancer status that may seem to contrast our results [28]. It is, however, challenging to compare two such different studies of which one rely on self-reported skincare product use collected years prior to breast cancer diagnosis and the other on paraben measurements in urine samples collected after breast cancer diagnosis. Clearly, Parada et al. showed that women with breast cancer experienced slightly higher concentrations of parabens compared to healthy controls, however reverse causation cannot be fully ruled out as the samples were donated after disease diagnosis.

Our results further suggest that use of skincare products does not increase the risk of endometrial cancer, an estrogen sensitive cancer type that have increased in incidence over the last 50 years in Norway [46]. In fact, compared to non−/light users of skincare products, moderate users experienced 23% decreased risk of endometrial cancer. However, due to the lack of linear trend across effect estimates and the lack of inverse association when modelling skincare product use in continuous scale and after multiple imputation, we consider this a random finding.

Use of PCPs is considered an important source of paraben exposure for humans [5, 12]. Several in vivo and in vitro studies have also demonstrated that parabens have the ability to interfere with the endocrine system and may increase the risk of breast cancer through various mechanisms [21, 22, 47, 48]. As a consequence, the use of parabens in consumer products within the European Union has been restricted and other biocides such as isothiazolinones have been introduced as replacements compounds. Lately, concern have been raised by dermatologists as these compounds poses much stronger sensitizing properties than parabens and an increasing incidence of allergic contact dermatitis is therefore expected [49]. As there is a lack of epidemiological evidence of the effects of parabens on human health, one may question whether replacing parabens with a more potent sensitizer is wise? There are however, several possible explanations for the disagreement between in vivo*/*in vitro studies and epidemiological studies that include i) the exposure classification in large scale epidemiological studies is challenging, as exposure usually is estimated from questionnaires. This limits the individual exposure assessment as detailed plasma concentration of EDs for each participant is not available. The questionnaire based approach has however been used for decades in nutritional epidemiology and if a proper validation study is performed, as in the our pervious study [24], the questionnaires will be able to rank individuals into broader categories based on their exposure to EDs from PCPs. ii) It is possible that humans have a different susceptibility to EDs compared to cell lines and experimental animals and that effects seen in in vivo and/or in vitro studies are not as pronounced among humans. Animal models will continue to provide important insight into the human biology, however there are also important differences between mice and men that needs to be addressed [50]. iii) The exposure measure in epidemiological studies may not necessarily cover exposure during etiological relevant periods. A large challenge when studying EDs in relations to hormone sensitive cancers is the limited knowledge about which period in life humans are most susceptible for ED exposure in relation to the outcome of interest. Recently, Harley et al. reported that urinary concentrations of parabens at age 9 were associated with earlier breast development, menarche and development of pubic hair [16]. They also found that prenatal triclosan concentrations were associated with earlier menarche. Thus, this study suggest that prenatal and peripubertal exposure to EDs from PCPs may modify important risk factors for breast and endometrial cancer, such as age at menarche. Many questionnaire-based epidemiological studies assess exposure during the previous year and assume that it reflect past exposure. However, as lifestyle and consumers products often change from puberty to adulthood, the exposure assessed later in life does not necessarily reflect exposure during etiologically sensitive time windows. Thus, due to the widespread use of cosmetics and skincare products among young women today, the precautionary principle of phasing out potentially harmful EDs may be favorable even though they may be replaced by compounds with stronger sensitizing properties. However, there is clearly a lack of epidemiological studies that assess the effect of ED exposure from PCPs during various life-phases and the subsequent risk of hormone related cancer.

The strengths of this study includes the large and national representative sample, the linkage to national registries that ensures complete information about death, emigration and cancer diagnosis and the detailed information about sociodemographic, reproductive and lifestyle variables that we included in our analyses. Our previous study of plasma concentrations of parabens in relation to reported use of skincare products confirmed that individuals classified as frequent or heavy users of skincare products experience elevated circulatory concentrations of methyl-, ethyl- and propyl parabens compared to the non-users [24]. Philippat et al. also confirmed a positive association between the number of PCPs used and increasing urinary paraben concentrations. They further suggested that questionnaires can be used to measure exposure to parabens [12]. However, skincare products are not only sources of parabens; they are sources of other EDs with different properties and mechanisms of action, individually and as mixtures. As we have not measured other EDs than parabens in plasma from skincare products users in NOWAC, we cannot be certain that those classified as frequent or heavy users also experienced the highest concentrations of other EDs such as phthalates, UV filters and triclosan that individually or combined may exert estrogenic activity.

We have previously assessed the change in use of skincare products over an eight year period. We found moderate agreement (weighted kappa: 0.52) and that almost 92% of women were classified into the same user group ±1 category [1]. Thus, the use of skincare products among these middle-aged women is a well-established habit that is relatively stable over time. Nevertheless, we cannot assume that the reported use of skincare products reflect exposure during childhood/puberty/adolescence. In fact, given the advanced age of the study participants, it is likely that their use of skincare products during childhood and adolescence were lower than what is common among children and adolescents today as the general Norwegian household economy was much weaker in the 1950’s and 60’s than nowadays and the availability to skincare products were much more limited. Caution should therefore be made if generalizing these results to younger birth cohorts (born 1960 and onwards).

The NOWAC questionnaires did not include questions about which part of the body that was covered in body lotion; therefore we assumed that one application of body lotion covered nearly the whole body (91%). This may be seen upon as a limitation as many women only use body lotion on arms and legs, suggesting that our measure “% skin covered in cream per day” may overestimate the exposure. However, we have previously shown that “% skin covered in cream per day” was strongly associated with plasma concentrations of parabens within the NOWAC cohort [24], and we believe that this measure better reflect blood concentrations of EDs from skincare product use than the frequency of use. Nevertheless, we conducted several additional analyses of reported usage frequencies of skincare products (Additional files 4 and 5). These analyses confirmed our main results, i.e. no increased risk of breast or endometrial cancer by frequent use of skincare products. Still, it is possible that some NOWAC participants over- or under-reported their usage frequencies of skincare products. As all information was collected before cancer diagnosis, this measurement error is likely non-differential. In our age-adjusted models, assuming non-differential, nonsystematic errors, misclassification would attenuate the HR of the higher skincare category, but the test for trend would be valid [51]. As we have several exposure categories and many confounding variables included in our multivariable models, non-differential misclassification can bias the estimates both towards and away from the null [51].

Our study was also unable to address whether the location of body lotion application was associated with breast or endometrial cancer risk. This may be important if dermal absorption of EDs on specific areas is more relevant for breast/endometrial carcinogenesis than continuously elevated systemic concentrations. Finally, we did not have information on antiperspirant and cosmetic use, which could be important sources of paraben exposure. Nevertheless, previous studies have called for epidemiological investigations in order to confirm or refute in vivo*/*in vitro findings regarding paraben exposure and the risk of breast cancer. Our finding that use of skincare products during mid-life was not associated with breast and endometrial cancer risk adds important knowledge to this research field.

## Conclusion

Our population-based prospective cohort study provides evidence that heavy use of skincare products, i.e. creaming the body up to two time per day during mid-life, does not increase the risk of pre- or postmenopausal breast cancer, ER+ or ER- breast cancer or endometrial cancer.

## Supplementary information

**Additional file 1.** Hazard ratios (HRs) and 95% confidence intervals (CIs) for the association between skincare product use and risk of cancer. Skincare product use modelled in continuous scale using restricted cubic spline transformations of “% skin covered in cream per day” with 4 knots.

**Additional file 2.** Hazard ratios (HRs) and 95% confidence intervals (CIs) for the associations between skincare product use and risk of pre- and postmenopausal breast cancer and endometrial cancer after multiple imputation by chained equations of missing values of included covariates.

**Additional file 3.** Hazard ratios (HRs) and 95% confidence intervals (CIs) for the associations between skincare product use and risk of estrogen receptor positive (ER+) and negative (ER-) breast cancer after multiple imputation by chained equations of missing values of included covariates.

**Additional file 4.** Hazard ratios (HRs) and 95% confidence intervals (CIs) for the associations between usage frequencies of body lotion and risk of pre- and postmenopausal breast cancer, endometrial cancer, ER+ and ER- breast cancer.

**Additional file 5.** Hazard ratios (HRs) and 95% confidence intervals (CIs) for the associations between usage frequencies of body lotion, hand cream and facial cream combined and risk of pre- and postmenopausal breast cancer, endometrial cancer, ER+ and ER- breast cancer.

## Data Availability

The datasets used and/or analyzed during the current study are available from the corresponding author on reasonable request.

## Abbreviations

ER+: Estrogen receptor positive
ER-: Estrogen receptor negative
HR: Hazard ratio
CI: Confidence interval
PCP: Personal care product
ED: Endocrine disruptor
NOWAC: the Norwegian Women And Cancer study
ICD-10: International Statistical Classification of Diseases, Injuries and Causes of Death Revision 10
MHT: Menopausal hormone therapy
BMI: Body mass index
OC: Oral contraceptives
IUD: Intrauterine device
SD: Standard deviation
