# Supplementary material for: Use of skincare products and risk of cancer of the breast and endometrium: a prospective cohort study
Source: Environ Health. 2019 Dec 3;18:105. doi: 10.1186/s12940-019-0547-6 (PMC6889352; doi:10.1186/s12940-019-0547-6)
Supplement: Supplementary file 1 — Additional file 1. Hazard ratios (HRs) and 95% confidence intervals (CIs) for the association between skincare product use and risk of cancer. Skincare product use modelled in continuous scale using restricted cubic spline transformations of “% skin covered in cream per day” with 4 knots. [file 12940_2019_547_MOESM1_ESM.docx]

Additional file 1: Hazard ratios (HRs) and 95% confidence intervals (CIs) for the association between skincare product use and risk of cancer. Skincare product use modelled in continuous scale using restricted cubic spline transformations of “% skin covered in cream per day” with 4 knots.

| % skin covered in cream per day | Premenopausal breast cancer ^a^, HR (95% CI) | Postmenopausal breast cancer ^b^, HR (95% CI) | Endometrial cancer ^c^, HR (95% CI) | ER+ breast cancer ^d^, HR (95% CI) | ER– breast cancer ^e^, HR (95% CI) |
| --- | --- | --- | --- | --- | --- |
| 0 | 1.00 | 1.00 | 1.00 | 1.00 | 1.00 |
| 25 | 1.04 (0.74, 1.44) | 0.86 (0.76, 0.99) | 0.85 (0.63, 1.15) | 0.88 (0.76, 1.01) | 0.77 (0.55, 1.08) |
| 50 | 1.10 (0.82, 1.48) | 0.86 (0.74, 0.99) | 0.85 (0.62, 1.17) | 0.88 (0.75, 1.02) | 0.76 (0.53, 1.08) |
| 75 | 1.12 (0.86, 1.45) | 0.90 (0.80, 1.02) | 0.90 (0.68, 1.18) | 0.92 (0.80, 1.04) | 0.83 (0.61, 1.12) |
| 100 | 1.09 (0.84, 1.41) | 0.88 (0.79, 1.00) | 0.89 (0.68, 1.16) | 0.91 (0.80, 1.04) | 0.83 (0.62, 1.11) |
| 125 | 1.05 (0.77, 1.45) | 0.84 (0.72, 0.97) | 0.85 (0.61, 1.18) | 0.87 (0.75, 1.01) | 0.80 (0.56, 1.14) |
| 150 | 1.01 (0.67, 1.54) | 0.79 (0.65, 0.96) | 0.82 (0.52, 1.29) | 0.84 (0.68, 1.02) | 0.76 (0.47, 1.24) |
| 175 | 0.98 (0.57, 1.67) | 0.75 (0.58, 0.97) | 0.78 (0.43, 1.43) | 0.80 (0.62, 1.05) | 0.73 (0.39, 1.40) |
| 200 | 0.94 (0.49, 1.82) | 0.71 (0.51, 0.98) | 0.75 (0.35, 1.61) | 0.77 (0.56, 1.08) | 0.70 (0.31, 1.59) |

^a^ Adjusted for maternal breast cancer history and alcohol intake. ^b^ Adjusted for body mass index, use of menopause hormone therapy, age at first birth and parity combined, maternal breast cancer history, physical activity and alcohol intake. ^c^ Adjusted for body mass index, use of oral contraceptives, use of intrauterine device, smoking and education. ^d^ Adjusted for body mass index, smoking, age at first birth and parity combined, alcohol intake, physical activity, menopausal status, maternal breast cancer history and use of menopause hormone therapy. ^e^ Adjusted for physical activity and maternal breast cancer history.
