# Supplementary material for: Use of skincare products and risk of cancer of the breast and endometrium: a prospective cohort study
Source: Environ Health. 2019 Dec 3;18:105. doi: 10.1186/s12940-019-0547-6 (PMC6889352; doi:10.1186/s12940-019-0547-6)
Supplement: Supplementary file 2 — Additional file 2. Hazard ratios (HRs) and 95% confidence intervals (CIs) for the associations between skincare product use and risk of pre- and postmenopausal breast cancer and endometrial cancer after multiple imputation by chained equations of missing values of included covariates. [file 12940_2019_547_MOESM2_ESM.docx]

Additional file 2. Hazard ratios (HRs) and 95 % confidence intervals (CIs) for the associations between skincare product use and risk of pre- and postmenopausal breast cancer and endometrial cancer after multiple imputation by chained equations of missing values of included covariates.

| User groups of skincare products per cancer type | Age-adjusted HR (95% CI) | Multivariable HR (95% CI) | p^trend^ |
| --- | --- | --- | --- |
| Premenopausal breast cancer^a^ |  |  |  |
| Non-/Light users | 1.00 | 1.00 | 0.25 |
| Moderate users | 1.04 (0.85,1.26) | 1.04 (0.85,1.27) |  |
| Frequent/heavy users | 1.12 (0.93,1.33) | 1.11 (0.93,1.33) |  |
| Postmenopausal breast cancer^b^ |  |  |  |
| Non-users | 1.14 (0.87,1.48) | 1.16 (0.89,1.51) | 0.34 |
| Light users | 1.00 | 1.00 |  |
| Moderate users | 0.98 (0.89,1.07) | 0.97 (0.88,1.06) |  |
| Frequent users | 0.99 (0.90,1.08) | 0.96 (0.88,1.05) |  |
| Heavy users | 0.83 (0.63,1.10) | 0.81 (0.61,1.08) |  |
| Endometrial cancer^c^ |  |  |  |
| Non-/Light users | 1.00 | 1.00 | 0.83 |
| Moderate users | 0.76 (0.62,0.93) | 0.84 (0.69,1.03) |  |
| Frequent/heavy users | 0.87 (0.73,1.03) | 1.01 (0.85,1.20) |  |

^a^ n=21 257, 666 cases. Multivariable adjusted for maternal breast cancer history and alcohol intake. ^b^ n= 106 328, 3118 cases. Multivariable adjusted for body mass index, use of menopause hormone therapy, age at first birth and parity combined, maternal breast cancer history, physical activity and alcohol intake. ^c^ n=95 577, 679 cases. Multivariable adjusted for body mass index, use of oral contraceptives, use of intrauterine device, smoking and education.
