# Supplementary material for: Use of skincare products and risk of cancer of the breast and endometrium: a prospective cohort study
Source: Environ Health. 2019 Dec 3;18:105. doi: 10.1186/s12940-019-0547-6 (PMC6889352; doi:10.1186/s12940-019-0547-6)
Supplement: Supplementary file 3 — Additional file 3. Hazard ratios (HRs) and 95% confidence intervals (CIs) for the associations between skincare product use and risk of estrogen receptor positive (ER+) and negative (ER-) breast cancer after multiple imputation by chained equations of missing values of included covariates. [file 12940_2019_547_MOESM3_ESM.docx]

Additional file 3: Hazard ratios (HR) and 95 % confidence intervals (CI) for the associations between skincare product use and risk of estrogen receptor positive (ER+) and negative (ER-) breast cancer after multiple imputation by chained equations of missing values of included covariates.

|  | ER+ |  | ER- |  |  |
| --- | --- | --- | --- | --- | --- |
| User groups of skincare products | Age-adjusted HR (95% CI) | Multivariable HR (95% CI)^a^ | Age-adjusted HR(95% CI) | Multivariable HR (95% CI)^c^ | p_heterogeneity_^c^ |
| Non-/light users | 1.00 | 1.00 | 1.00 | 1.00 |  |
| Moderate users | 0.97 (0.88,1.07) | 0.97 (0.88,1.07) | 0.86 (0.67,1.10) | 0.88 (0.69,1.12) | 0.45 |
| Frequent/heavy users | 0.98 (0.89,1.07) | 0.97 (0.88,1.06) | 0.92 (0.74,1.15) | 0.96 (0.77,1.19) | 0.94 |

^a^ n=106 978, 2868 cases. Multivariable adjusted for body mass index, smoking, age at first birth and parity combined, alcohol intake, physical activity, menopausal status, maternal breast cancer history and use of menopause hormone therapy. ^b^ n= 106 978, 454 cases. Multivariable adjusted for physical activity and maternal breast cancer history. ^c^ Test for difference by breast cancer subtypes.
