# Supplementary material for: Use of skincare products and risk of cancer of the breast and endometrium: a prospective cohort study
Source: Environ Health. 2019 Dec 3;18:105. doi: 10.1186/s12940-019-0547-6 (PMC6889352; doi:10.1186/s12940-019-0547-6)
Supplement: Supplementary file 4 — Additional file 4. Hazard ratios (HRs) and 95% confidence intervals (CIs) for the associations between usage frequencies of body lotion and risk of pre- and postmenopausal breast cancer, endometrial cancer, ER+ and ER- breast cancer. [file 12940_2019_547_MOESM4_ESM.docx]

Additional file 4: Hazard ratios (HRs) and 95 % confidence intervals (CIs) for the associations between usage frequencies of body lotion and risk of pre- and postmenopausal breast cancer, endometrial cancer, ER+ and ER- breast cancer.

| Frequency of body lotion use per cancer type | n | Cancer cases | Age-adjusted HR (95% CI) | Multivariable HR (95% CI) | p_trend_ | p_heterogeneity_^a^ |
| --- | --- | --- | --- | --- | --- | --- |
| Premenopausal breast cancer^b^ |  |  |  |  |  |  |
| Never/seldom | 2977 | 85 | 1.00 | 1.00 | 0.55 |  |
| 1-4 times/month | 5127 | 158 | 1.08 (0.83,1.4) | 1.07 (0.82,1.39) |  |  |
| 2-6 times/week | 6578 | 223 | 1.19 (0.93,1.53) | 1.17 (0.91,1.50) |  |  |
| 1-≥2 times/day | 4741 | 145 | 1.08 (0.83,1.42) | 1.06 (0.81,1.39) |  |  |
| Postmenopausal breast cancer^c^ |  |  |  |  |  |  |
| Never/seldom | 12762 | 380 | 1.00 | 1.00 | 0.32 |  |
| 1-4 times/month | 21866 | 615 | 0.94 (0.83,1.07) | 0.93 (0.82,1.06) |  |  |
| 2-6 times/week | 32178 | 934 | 0.96 (0.85,1.08) | 0.94 (0.84,1.07) |  |  |
| 1-≥2 times/day | 23844 | 686 | 0.95 (0.84,1.08) | 0.92 (0.81,1.05) |  |  |
| Endometrial cancer^d^ |  |  |  |  |  |  |
| Never/seldom | 9682 | 82 | 1.00 | 1.00 | 0.51 |  |
| 1-4 times/month | 16604 | 135 | 0.96 (0.73,1.27) | 1.07 (0.81,1.41) |  |  |
| 2-6 times/week | 23415 | 143 | 0.71 (0.54,0.93) | 0.84 (0.64,1.11) |  |  |
| 1-≥2 times/day | 17521 | 121 | 0.80 (0.6,1.06) | 1.00 (0.75,1.33) |  |  |
| ER+ breast cancer^e^ |  |  |  |  |  |  |
| Never/seldom | 12498 | 341 | 1.00 | 1.00 | 0.51 |  |
| 1-4 times/month | 21497 | 560 | 0.95 (0.83,1.09) | 0.95 (0.83,1.09) |  | 0.35 |
| 2-6 times/week | 31682 | 846 | 0.97 (0.86,1.10) | 0.97 (0.86,1.10) |  | 0.27 |
| 1-≥2 times/day | 23452 | 615 | 0.95 (0.83,1.09) | 0.94 (0.82,1.08) |  | 0.42 |
| ER- breast cancer^f^ |  |  |  |  |  |  |
| Never/seldom | 13216 | 69 | 1.00 | 1.00 | 0.30 |  |
| 1-4 times/month | 22609 | 95 | 0.80 (0.59,1.09) | 0.81 (0.59,1.10) |  |  |
| 2-6 times/week | 33238 | 135 | 0.79 (0.59,1.05) | 0.81 (0.61,1.09) |  |  |
| 1-≥2 times/day | 24748 | 100 | 0.78 (0.57,1.06) | 0.82 (0.60,1.12) |  |  |

HR: hazard ratio; CI: confidence interval; ER: estrogen receptor; ^a^Test of difference in association between ER+ and ER- breast cancer ^b^ Multivariable adjusted for maternal breast cancer history and alcohol intake. ^c^ Multivariable adjusted for body mass index, use of menopause hormone therapy, age at first birth and parity combined, maternal breast cancer history, physical activity and alcohol intake; ^d^ Multivariable adjusted for body mass index, use of oral contraceptives, use of intrauterine device, smoking and education; ^e^ Multivariable adjusted for body mass index, smoking, age at first birth and parity combined, alcohol intake, physical activity, menopausal status, maternal breast cancer history and use of menopause hormone therapy; ^f^ Multivariable adjusted for physical activity and maternal breast cancer history.
