# Supplementary material for: Use of skincare products and risk of cancer of the breast and endometrium: a prospective cohort study
Source: Environ Health. 2019 Dec 3;18:105. doi: 10.1186/s12940-019-0547-6 (PMC6889352; doi:10.1186/s12940-019-0547-6)
Supplement: Supplementary file 5 — Additional file 5. Hazard ratios (HRs) and 95% confidence intervals (CIs) for the associations between usage frequencies of body lotion, hand cream and facial cream combined and risk of pre- and postmenopausal breast cancer, endometrial cancer, ER+ and ER- breast cancer. [file 12940_2019_547_MOESM5_ESM.docx]

Additional file 5: Hazard ratios (HRs) and 95 % confidence intervals (CIs) for the associations between usage frequencies of body lotion, hand cream and facial cream combined^a^ and risk of pre- and postmenopausal breast cancer, endometrial cancer, ER+ and ER- breast cancer.

| Frequency of skincare product use per cancer type | n | Cancer cases | Age-adjusted HR (95% CI) | Multivariable HR (95% CI) | p_trend_ | p_heterogeneity_^b^ |
| --- | --- | --- | --- | --- | --- | --- |
| Premenopausal breast cancer^c^ |  |  |  |  |  |  |
| Low (0-5) | 2064 | 53 | 1.00 | 1.00 | 0.94 |  |
| Moderate low (6-10) | 5526 | 185 | 1.31 (0.97,1.78) | 1.29 (0.95,1.76) |  |  |
| Moderate high (11-14) | 6716 | 218 | 1.26 (0.93,1.7) | 1.24 (0.91,1.67) |  |  |
| High (15-18) | 4701 | 137 | 1.15 (0.84,1.58) | 1.12 (0.82,1.54) |  |  |
| Postmenopausal breast cancer^d^ |  |  |  |  |  |  |
| Low (0-5) | 8828 | 255 | 1.00 | 1.00 | 0.02 |  |
| Moderate low (6-10) | 23168 | 696 | 1.03 (0.89,1.19) | 1.01 (0.88,1.17) |  |  |
| Moderate high (11-14) | 32487 | 919 | 0.95 (0.83,1.10) | 0.94 (0.81,1.08) |  |  |
| High (15-18) | 23833 | 656 | 0.92 (0.80,1.07) | 0.89 (0.77,1.03) |  |  |
| Endometrial cancer^e^ |  |  |  |  |  |  |
| Low (0-5) | 6639 | 56 | 1.00 | 1.00 | 0.45 |  |
| Moderate low (6-10) | 17569 | 142 | 0.95 (0.70,1.29) | 1.11 (0.82,1.52) |  |  |
| Moderate high (11-14) | 23842 | 168 | 0.81 (0.60,1.09) | 1.02 (0.75,1.39) |  |  |
| High (15-18) | 17436 | 108 | 0.71 (0.51,0.98) | 0.96 (0.69,1.33) |  |  |
| ER+ breast cancer^f^ |  |  |  |  |  |  |
| Low (0-5) | 8620 | 226 | 1.00 | 1.00 | 0.05 |  |
| Moderate low (6-10) | 22806 | 649 | 1.07 (0.92,1.25) | 1.07 (0.91,1.24) |  | 0.11 |
| Moderate high (11-14) | 31962 | 816 | 0.95 (0.82,1.11) | 0.95 (0.82,1.11) |  | 0.69 |
| High (15-18) | 23472 | 596 | 0.95 (0.81,1.1) | 0.93 (0.80,1.09) |  | 0.20 |
| ER- breast cancer^g^ |  |  |  |  |  |  |
| Low (0-5) | 9139 | 48 | 1.00 | 1.00 | 0.20 |  |
| Moderate low (6-10) | 23944 | 97 | 0.76 (0.54,1.08) | 0.78 (0.55,1.11) |  |  |
| Moderate high (11-14) | 33571 | 148 | 0.84 (0.60,1.16) | 0.88 (0.64,1.23) |  |  |
| High (15-18) | 24700 | 87 | 0.67 (0.47,0.95) | 0.72 (0.51,1.03) |  |  |

HR: hazard ratio; CI: confidence interval; ER+: estrogen receptor; ^a^A composite variable was computed by summarizing reported usage frequencies of body lotion, facial cream and hand cream. Zero corresponds to no use of any skincare products whereas 18 corresponds to use of body lotion, hand cream and facial cream two times per day or more; ^b^Test of difference in association between ER+ and ER- breast cancer ^c^ Multivariable adjusted for maternal breast cancer history and alcohol intake. ^d^ Multivariable adjusted for body mass index, use of menopause hormone therapy, age at first birth and parity combined, maternal breast cancer history, physical activity and alcohol intake; ^e^ Multivariable adjusted for body mass index, use of oral contraceptives, use of intrauterine device, smoking and education; ^f^ Multivariable adjusted for body mass index, smoking, age at first birth and parity combined, alcohol intake, physical activity, menopausal status, maternal breast cancer history and use of menopause hormone therapy; ^g^ Multivariable adjusted for physical activity and maternal breast cancer history.
